# A combination of selenium and *Bacillus subtilis* improves the quality and flavor of meat and slaughter performance of broilers

**DOI:** 10.3389/fvets.2023.1259760

**Published:** 2023-11-08

**Authors:** Jihong Dong, Huiling Qiu, Shansong Gao, Lele Hou, Huawei Liu, Lianqin Zhu, Fu Chen

**Affiliations:** ^1^Laboratory of Animal Nutrition Metabolic and Poisoning Diseases, College of Veterinary Medicine, Qingdao Agricultural University, Qingdao, Shandong, China; ^2^Haidu College, Qingdao Agricultural University, Laiyang, Shandong, China; ^3^College of Animal Science and Technology, Qingdao Agricultural University, Qingdao, Shandong, China

**Keywords:** selenium, *Bacillus subtilis*, slaughter performance, meat quality, broiler

## Abstract

This study aimed to investigate the effects of the combination of selenium and *Bacillus subtilis* (Se-BS) on the quality and flavor of meat and slaughter performance of broilers. A total of 240 one-day-old Arbor Acres broilers were randomly allotted to four treatments of a basal diet supplemented with no selenium (control), sodium selenite (SS), BS, or Se-BS and raised for 42 days. Compared with the control group, Se-BS significantly increased the carcass weight, the half-eviscerated weight, the completely eviscerated weight, the carcass rate, and redness in broiler muscles; improved the antioxidant state by increasing glutathione peroxidase (GPx) and glutathione S-transferase activities, the total antioxidant capacity, and *GPx-1* and thioredoxin reductase 1 messenger RNA (mRNA) levels; promoted biological activity by increasing the contents of glutamate, phenylalanine, lysine, and tyrosine; and increased Se and five types of nitrogenous volatile substances in muscles. On the other hand, Se-BS treatment decreased the shear force, drip loss, and the malondialdehyde, glutathione, and lead contents in muscles. Se-BS exerted a better effect on slaughter performance, the physicochemical quality of meat, the redox status, the amino acid contents, the trace element contents, and volatile substances compared with SS and BS. In conclusion, Se-BS had a positive effect on the quality and flavor of meat and slaughter performance of broilers, suggesting that Se-BS may be a beneficial feed additive.

## 1. Introduction

Chicken meat products are highly consumed. Improving the quality of broiler meat would help to promote its consumption and to increase the economic benefits of broiler breeding. Many factors affect meat quality in broilers, among which the addition of low doses of trace elements has a key impact on the maintenance and improvement of chicken quality (1–3). In particular, selenium (Se) is an important part of antioxidant enzymes such as glutathione peroxidase (GPx) and superoxide dismutase (SOD) in broilers (4). A sufficient supply of Se in broiler feed can improve the antioxidant capacity, ensure the integrity and functioning of the cell membrane, alleviate lipid peroxidation of unsaturated fatty acids, and slow myoglobin oxidation in muscles, all of which improve the color of chicken meat and prolong its shelf life (1, 5). Moreover, Se deficiency leads to white muscle disease, with muscle that appears pale and fluffy and with an exudate (6, 7). China is one of the regions with the greatest Se deficiency in the world, the Se-deficient regions account for 72% of the country’s area (8). Se supplementation is of great significance to promote animal growth and human health (1, 9). Usually, sodium selenite (SS), the representative inorganic form of Se, is added to feed to meet the needs of animals, but the absorption and utilization rates of SS are low (10, 11). Organic Se has a higher absorption rate and biosafety than SS. Therefore, the development and application of highly efficient organic Se sources has been a hotspot for livestock and poultry (5, 12).

Probiotics, as active microorganisms, that have been identified beneficial to the host. They can colonize the gut and reproductive system of humans and improve the host’s microecological balance (13, 14). *Bacillus subtilis* (BS) is a representative probiotic. Of note, BS is not affected by various enzymes in the gastrointestinal tract and helps to promote stress resistance and growth performance (15, 16). BS has become an ideal substitutes for antibiotics, and recent studies have confirmed that BS was an ideal Se supplement. This microorganism contains a rich array of metabolic enzymes that can convert inorganic Se into highly active organic Se. Hence, BS plays a dual role as a probiotic and a means to increase organic Se in the host (17–19), including modulating mercury (Hg)-induced intestinal microbial changes, reducing the abundance of *Aeromonas* and inflammation in fish, and modifying the ileal bacterial composition (14, 18). However, there are few studies that have evaluated whether a combination of Se and *Bacillus subtilis* (Se-BS) can improve meat quality in livestock and poultry. Therefore, this study evaluated the effects of Se-BS on the meat quality of Arbor Acres broilers to provide a reference for the future application of Se-BS in poultry.

## 2. Materials and methods

### 2.1. Animals and experimental treatments

All animal experiments were reviewed and approved by the Animal Care and Use Committee of Qingdao Agricultural University in accordance with laboratory animal guidelines (GB/T35892-2018, National Standards of the People’s Republic of China). A total of 240 one-day-old Arbor Acres broilers weighing 43–46 g, were purchased from Qingdao Aote Poultry Breeding Company (Qingdao, China). They were randomly divided into four groups with six replicate pens of 10 chickens each. Chickens were assigned to the following treatments: (1) the control group fed a basal diet (0.015 mg Se/kg) that Se was not added additionally for 42 days; (2) the SS group fed a basal diet supplemented with 0.30 mg Se/kg diet in the form of SS for 42 days; (3) the BS group fed a basal diet supplemented with BS (3 × 10^9^ colony-forming units [CFU] per g diet) for 42 days; and (4) the Se-BS group fed a basal diet supplemented with 0.30 mg Se/kg diet and BS (3 × 10^9^ CFU per g diet) for 42 days. The basal diet was formulated to approximately meet the nutrient requirements for broilers (NRC, 1994). Chickens had *ad libitum* access to food and water. The basal diet formulation and composition are shown in Table 1. During the 42-day experiment, all chickens were housed in a closed and ventilated building and provided with continuous light. Room temperature was controlled at 32–35°C for 3 days, and then gradually reduced by 3°C/week until it reached 24°C, where it was maintained for the remainder of the experiment. A previous study from our group had revealed that the average daily feed intake was similar in both groups, but the feed conversion ratio was lower for chickens fed SS, BS, and Se-BS (20).

**Table 1:** Formulation and composition of the experimental diets.

Ingredient (%)	1–21 d	22–42 d	Composition (g/kg, DM)	1–21 d	22–42 d
Corn	60.00	64.50	Metabolizable energy (MJ/kg)	12.15	13.06
Corn gluten meal (50% CP)	5.00	3.00	Crude protein	22.99	20.00
Wheat bran	0.00	2.00	Calcium	1.00	0.90
Soybean meal	27.50	25.00	Total phosphorus	0.45	0.35
Fishmeal (55.5% CP)	3.40	1.60	Methionine	0.50	0.38
Stone powder	1.20	1.40	Lysine	1.10	1.00
Salt	0.30	0.30	As (μg/kg)	–	1.02
Calcium bicarbonate	1.50	1.20	Cr (μg/kg)	17.32	16.30
Methionine	0.10	0.00	Cd (μg/kg)	6.50	6.02
Premix^a^	1.00	1.00	Hg (μg/kg)	0.03	0.04
			Pb (μg/kg)	6.07	6.46

^a^The vitamins provided (per kg diet): vitamin A 1500 IU, vitamin D3 200 IU, vitamin E 20 IU, vitamin K 0.5 mg, vitamin B1 22 mg, vitamin B2 8.5 mg, vitamin B12 0.2 mg, folicin 0.55 mg, niacin 0.55 mg, pantothenic acid 10.0 mg, copper 8.0 mg, zinc 40.0 mg, iron 80 mg, iodine 0.35 mg, manganese 60.0 mg, and choline 500 mg.

### 2.2. Slaughter performance

On day 42, all chickens were fasted for 12 h. The total body weight (BW) was determined for each replicate pen of 10 chickens, and then the average BW was calculated. Five chickens were randomly selected from each replicate pen, with a total of 30 chickens in each group; they were euthanized by an intravenous injection of sodium pentobarbital (50 mg/kg of body weight) into the wing vein and slaughtered (21). The carcass weight, the half-eviscerated weight, the fully eviscerated weight, the carcass rate, the complete evisceration rate, and the indexes of the spleen, thymus, bursa of fabricius, liver, and glandular stomach were determined.

### 2.3. Sample collection and preparation

The left breast meat (pectoralis muscle) and leg meat (thigh muscle) of each chicken was collected to determine physicochemical characteristics. Fresh pectoralis muscle (not less than 1 g per chicken) was collected and stored in liquid nitrogen to determine messenger RNA (mRNA) levels of antioxidant enzyme genes. The right pectoral muscle was placed in a polyethylene bag and frozen at −70°C to analyze the redox status, amino acids, volatile substances, and trace elements.

### 2.4. Determination of physicochemical characteristics

The meat color (lightness, redness, and yellowness) was evaluated by using a CR410 Chroma Meter (Konica Minolta Sensing Singapore Pte Ltd., Singapore). The pH was measured with a digital pH meter (pH-STAR; Matthäus GmbH & Co. KG, Eckelsheim, Germany). Shear force was measured with a digital muscle tenderness meter (C-LM3, Tenovo International Co., Ltd., Beijing, China). Drip loss was estimated by the suspension method. The dry matter content was measured with the drying method, and the cooked meat rate was measured with the boiling method.

### 2.5. Determination of the redox status

The protein content, the total antioxidant capacity (T-AOC), the malondialdehyde (MDA) and reduced glutathione (GSH) levels, and the GPx and glutathione S-transferase (GST) activities in muscles were analyzed according to the manufacturer’s instructions for the kits (Nanjing Jiancheng Bioengineering Institute, Nanjing, China). The mRNA levels of *GPx-1* and thioredoxin reductase 1 (*TR-1*) in the muscles were measured by using real-time quantitative reverse-transcription polymerase chain reaction (qRT-PCR) based upon SYBR Green I (1, 22, 23). Total RNA was extracted with the TRIzol Reagent (Sangon Biotech Co., Ltd., Shanghai, China) and complementary DNA (cDNA) was synthesized according to the instructions of the AMV First Strand cDNA Synthesis Kit (Sangon Biotech Co., Ltd.). The specific primers for gene expression were designed based on *Gallus gallus* sequences (Table 2). The *β-actin* housekeeping gene was used as an internal control. The qRT-PCR was performed with an ABI Real-time PCR System (StepOnePlus^™^; Applied Biosystems, Waltham, MA, USA). The mRNA levels of genes were calculated with the 2^-△△Ct^ method.

**Table 2:** Primer sequences.

Gene name	Primers (5′ to 3′)	Length
*β-actin*	Forward: agtgtctttttgtatcttccgcc Reverse: ccacatactggcactttactccta	147 bp
*GPx-1*	Forward: tctacctggtaactttcgagcaa Reverse: cctttattgcagagcctcctt	147 bp
*TR-1*	Forward: tcaagaatgtcaccgcaagtt Reverse: cacgcagataacatccccaat	129 bp

### 2.6. Analysis of amino acids and volatile substances

The amino acid contents were determined by using an amino acid analyzer (Hitachi, Japan), including glutamate (GLU), phenylalanine (PHE), lysine (LYS), and tyrosine (TYR). The contents of essential amino acids (EAA), umami amino acids (UAA), and total amino acids (TAA) were calculated. The concentrations of volatile substances were detected with gas chromatography (Agilent 6890N, USA).

### 2.7. Analysis of trace elements

Meat samples (0.5 g) were prepared, mixed with 10 mL of a mixed-acid solution (HNO_3_:HClO_4_ = 4:1), and digested on an electric heating plate. The concentration of trace elements, including Se, zinc (Zn), chromium (Cr), arsenic (As), Hg, cadmium (Cd), and lead (Pb), was determined with an atomic fluorescence spectrometer (AFS-9330, Beijing Titan Instruments Co., Ltd., Beijing, China).

### 2.8. Statistical analysis

All data were analyzed using SPSS Statistics 22.0 (IBM Corporation, Armonk, NY, USA) and tested for normality. Results are reported as mean ± standard deviation (SD). All data were analyzed using one-way analysis of variance (ANOVA) to compare means and the multivariate analysis of the generalized linear model procedure. The least significant difference (LSD) tests were used to determine differences between means. When variances were not homogeneous, data were analyzed through the non-parametric Kruskal–Wallis test followed by the Student–Newman–Keuls (SNK) test. The pen was defined as the experimental unit for statistical analysis, and all calculations were based on pen averages. For all tests, *p* < 0.05 was considered to be statistically significant.

## 3. Results

### 3.1. Effects of Se-BS on slaughter performance

**Figure 1:**
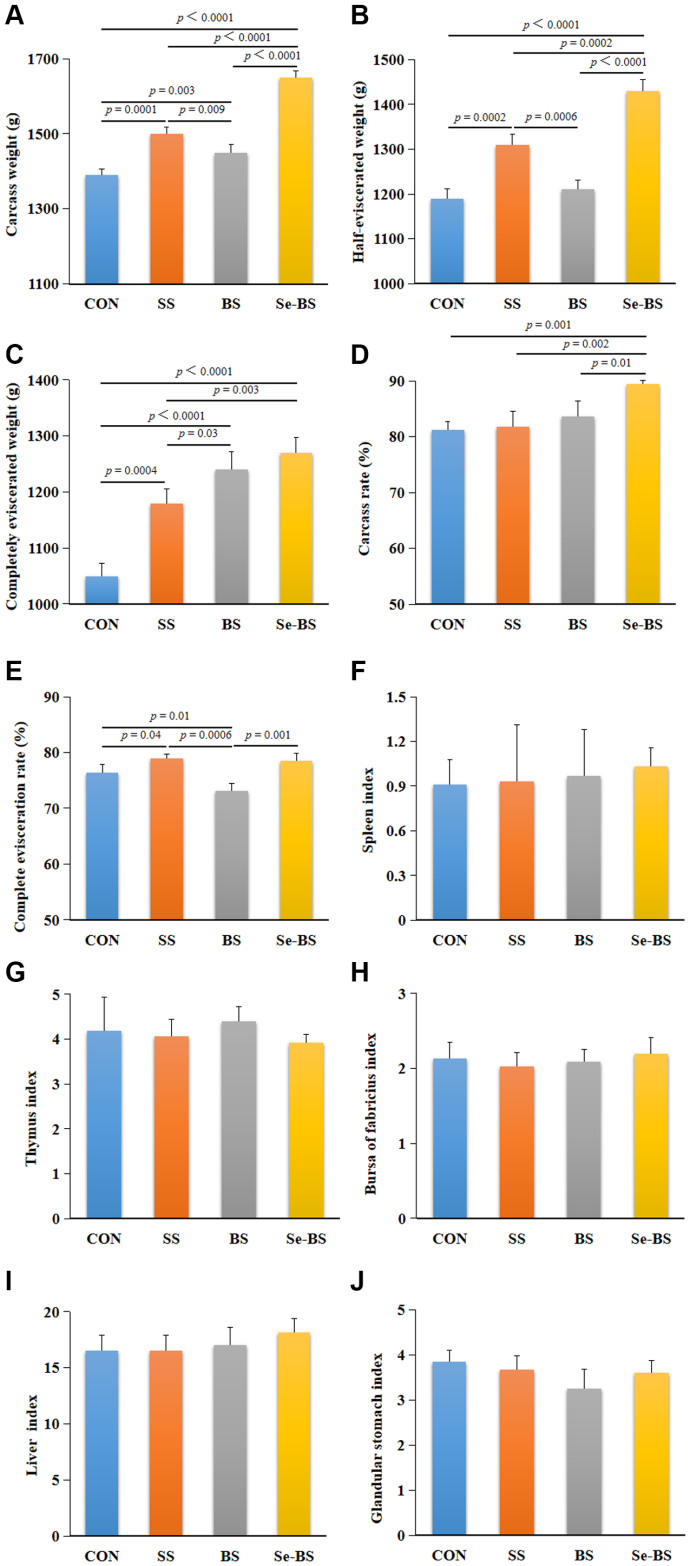
The effects of dietary supplementation with selenium (Se) and *Bacillus subtilis* (BS) on slaughter performance and organ indices of broilers. **(A)** The carcass weight, **(B)** the half-eviscerated weight, **(C)** the completely eviscerated weight, **(D)** the carcass rate, **(E)** the complete evisceration rate, **(F)** the spleen index, **(G)** the thymus index, **(H)** the bursa of fabricius index, **(I)** the liver index, and **(J)** the glandular stomach index. The data are presented as the mean ± standard deviation. *P*-values indicate group differences.

The effects of SS, BS, and Se-BS on slaughter performance of broilers are shown in Figure 1. Compared with the control group, the carcass weight (*p* = 0.0001, Figure 1A), the half-eviscerated weight (*p* = 0.0002, Figure 1B), the completely eviscerated weight (*p* = 0.0004, Figure 1C), and complete evisceration rate (*p* = 0.04, Figure 1E) were significantly increased in SS group. Compared with the control group, the carcass weight (*p* = 0.003, Figure 1A), and the completely eviscerated weight (*p* < 0.0001, Figure 1C) were significantly increased in BS group. However, the carcass weight (*p* < 0.0001, Figure 1A), the half-eviscerated weight (*p* < 0.0001, *p* = 0.0002, Figure 1B), the completely eviscerated weight (*p* < 0.0001, *p* = 0.0003, Figure 1C), the carcass rate (*p* = 0.001, *p* = 0.002, *p* = 0.01, Figure 1D), and the complete evisceration rate (*p* = 0.001) were significantly increased in Se-BS group compared with the control group, SS group or BS group. There were no significant differences between the four treatments regarding the organ indices (Figures 1F–J).

### 3.2. Effects of Se-BS on meat physicochemical quality

**Figure 2:**
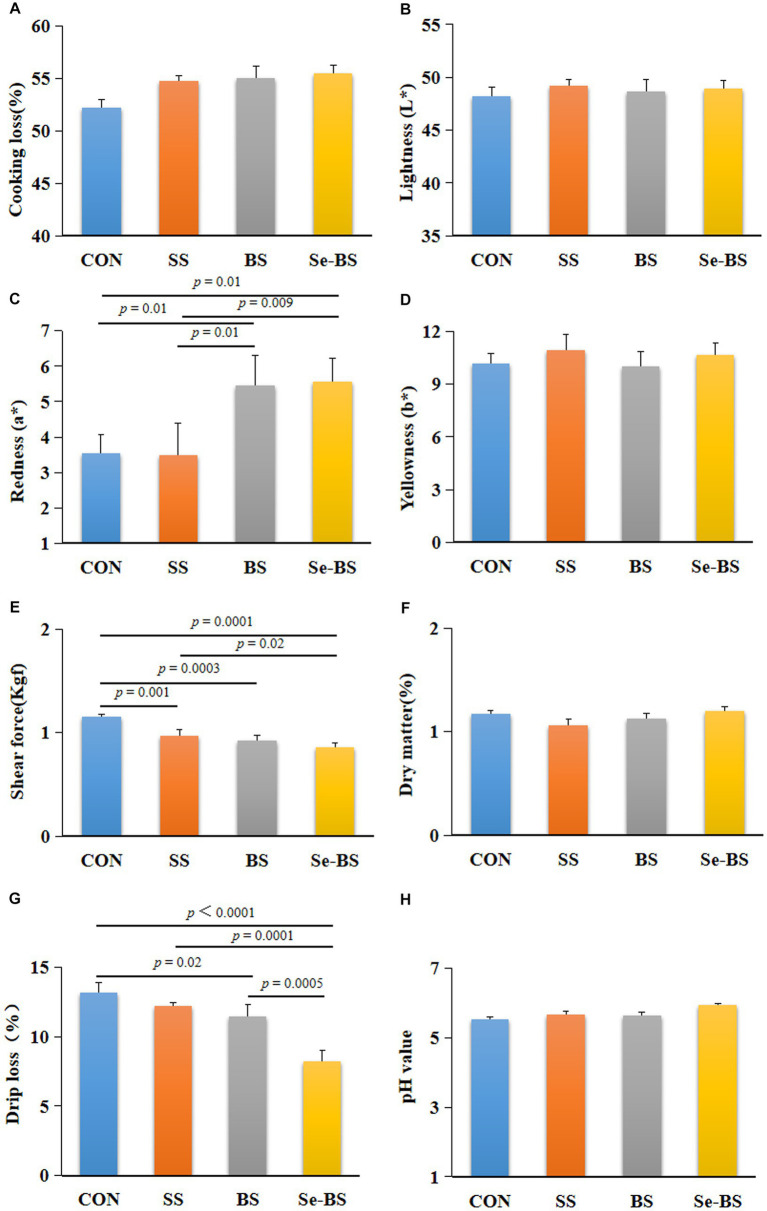
The effects of dietary supplementation with selenium (Se) and *Bacillus subtilis* (BS) on the physicochemical quality of the pectoral muscle in broilers. **(A)** Cooking loss, **(B)** meat color: lightness, **(C)** meat color: redness, **(D)** meat color: yellowness, **(E)** shear force, **(F)** dry matter, **(G)** drip loss, and **(H)** pH value. The data are presented as the mean ± standard deviation. *P*-values indicate group differences.

**Figure 3:**
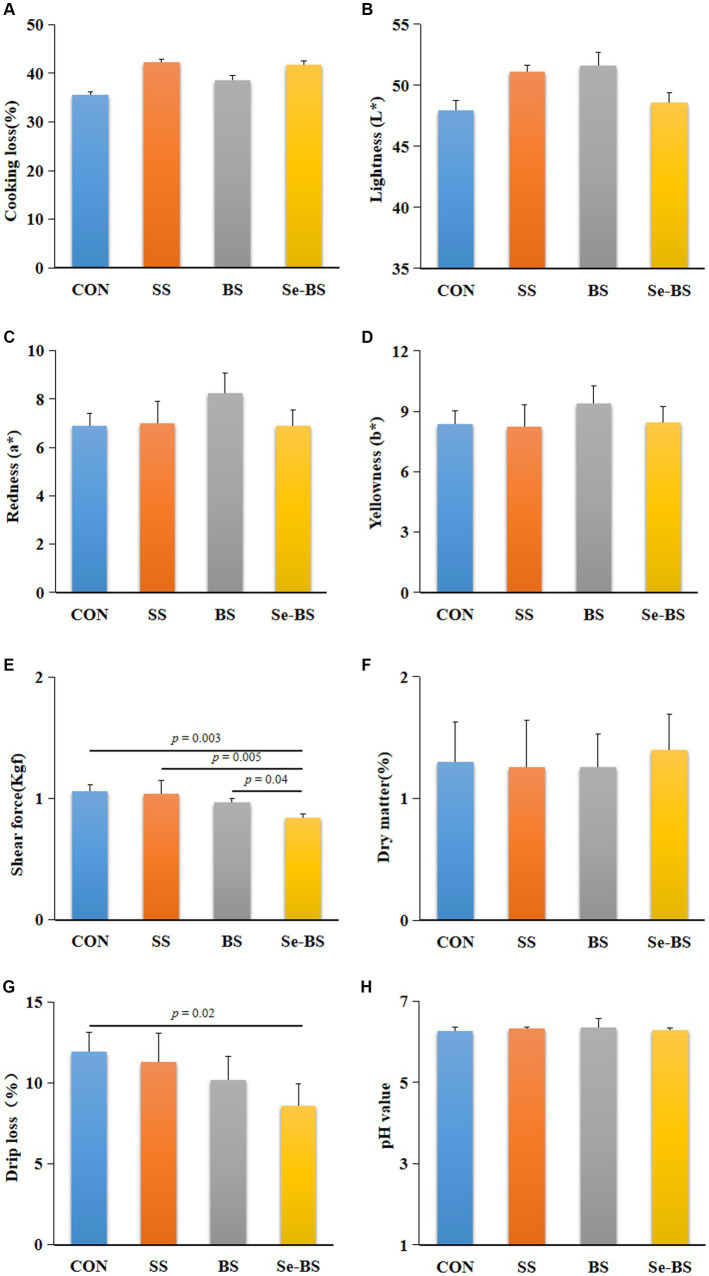
The effects of dietary supplementation with selenium (Se) and *Bacillus subtilis* (BS) on the physicochemical quality of the leg muscle in broilers. **(A)** Cooking loss, **(B)** meat color: lightness, **(C)** meat color: redness, **(D)** meat color: yellowness, **(E)** shear force, **(F)** dry matter, **(G)** drip loss, and **(H)** pH value. The data are presented as the mean ± standard deviation. *P*-values indicate group differences.

The effects of Se-BS on meat physicochemical quality are presented in Figures 2, 3. Compared with the control group, the redness of the pectoral muscle were significantly increased in BS and Se-BS groups (*p* = 0.01, Figure 2C); the shear force of the pectoral muscle was significantly decreased in SS (*p* = 0.001), BS (*p* = 0.0003), and Se-BS (*p* = 0.0001, Figure 2E) groups, the shear force of the leg muscle was significantly decreased in Se-BS (*p* = 0.003, Figure 3E) groups; the drip loss of the pectoral muscle was significantly decreased in BS (*p* = 0.02) and Se-BS groups (*p* < 0.0001, Figure 2G), the drip loss of the leg muscle was significantly decreased in Se-BS group (*p* = 0.02, Figure 3G).

Compared with the SS group, the redness of the pectoral muscle was significantly increased in BS group (*p* = 0.01, Figure 2C), Se-BS significantly increased the redness of the pectoral muscle (*p* = 0.009, Figure 2C) and decreased the shear force of the pectoral (*p* = 0.02, Figure 2E) and leg muscles (*p* = 0.005, Figure 3E). Finally, compared with the BS group, the drip loss of the pectoral muscle (*p* = 0.0005, Figure 2G) and the shear force of the leg muscle (*p* = 0.04, Figure 3E) was significantly decreased in Se-BS groups.

### 3.3. Effects of Se-BS on the redox status

**Figure 4:**
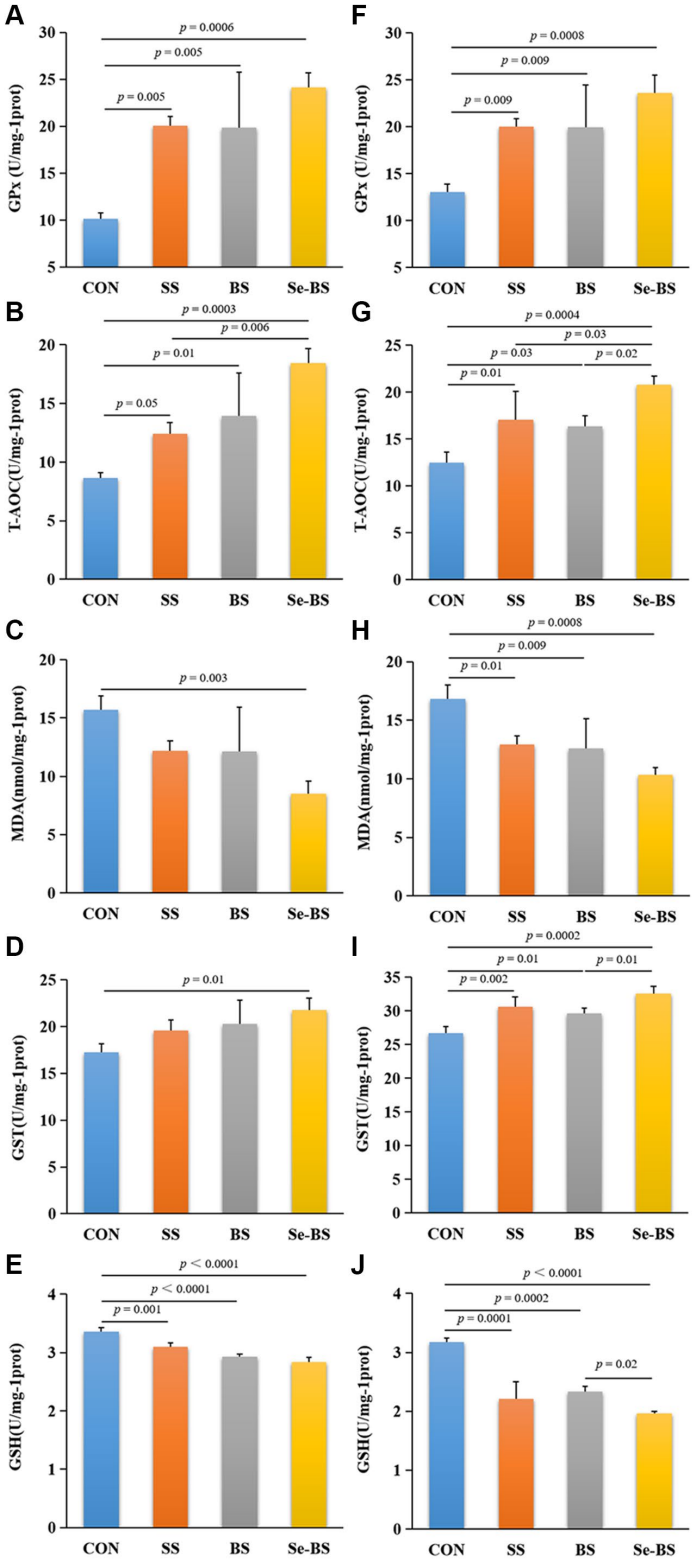
The effects of dietary supplementation with selenium (Se) and *Bacillus subtilis* (BS) on the antioxidant status of muscles in broilers. **(A)** Glutathione peroxidase (GPx) activity, **(B)** the total antioxidant capacity (T-AOC), **(C)** the malondialdehyde (MDA) content, **(D)** glutathione S-transferase (GST) activity, and **(E)** the reduced glutathione (GSH) content in the pectoral muscle; **(F)** GPx activity, **(G)** T-AOC, **(H)** the MDA content, **(I)** GST activity, and **(J)** the GSH content in the leg muscle. The data are presented as the mean ± standard deviation. *P*-values indicate group differences.

Effects of Se-BS on the redox status of the meat were analyzed and shown in Figure 4. Compared with the control group, GPx activity and T-AOC in the pectoral (*p* = 0.005, Figure 4A; *p* = 0.05, Figure 4B) and leg muscles (*p* = 0.009, Figure 4F; *p* = 0.01, Figure 4G) were significantly increased in SS group. Furthermore, the GST activity in the leg muscle was also significantly increased in SS group (*p* = 0.002, Figure 4I). Contents of MDA in the leg muscles (*p* = 0.01, Figure 4H) and GSH in the pectoral and leg muscles (*p* = 0.001, Figure 4E; *p* = 0.0001, Figure 4J) were significantly decreased. BS significantly increased the GPx activities (*p* = 0.005, Figure 4A; *p* = 0.009, Figure 4F) and T-AOC (*p* = 0.01, Figure 4B; *p* = 0.03, Figure 4G) in the pectoral and leg muscles, and GST (*p* = 0.01, Figure 4I) activities in the leg muscle. And BS significantly decreased the MDA content (*p* = 0.009, Figure 4H) in the leg muscle and the GSH (*p* < 0.0001, Figure 4E; *p* = 0.0002, Figure 4J) content in the pectoral and leg muscles. Se-BS significantly increased the GPx (*p* = 0.0006, Figure 4A; *p* = 0.0008, Figure 4F) and GST (*p* = 0.01, Figure 4D; *p* = 0.0002, Figure 4I) activities and T-AOC (*p* = 0.0003, Figure 4B; *p* = 0.0004, Figure 4G), and decreased the MDA (*p* = 0.003, Figure 4C; *p* = 0.0008, Figure 4H) and GSH contents (*p* < 0.0001, Figures 4E,J) in the pectoral and leg muscles. Compared with the SS group, the T-AOC in the pectoral muscle (*p* = 0.006, Figure 4B) as well as T-AOC in the leg muscle (*p* = 0.03, Figure 4G) were significantly increased in Se-BS group. Compared with the BS group, Se-BS significantly increased T-AOC (*p* = 0.02, Figure 4G) and the GST activity (*p* = 0.01, Figure 4I), and decreased the GSH contents (*p* = 0.02, Figure 4J) in the leg muscle.

**Figure 5:**
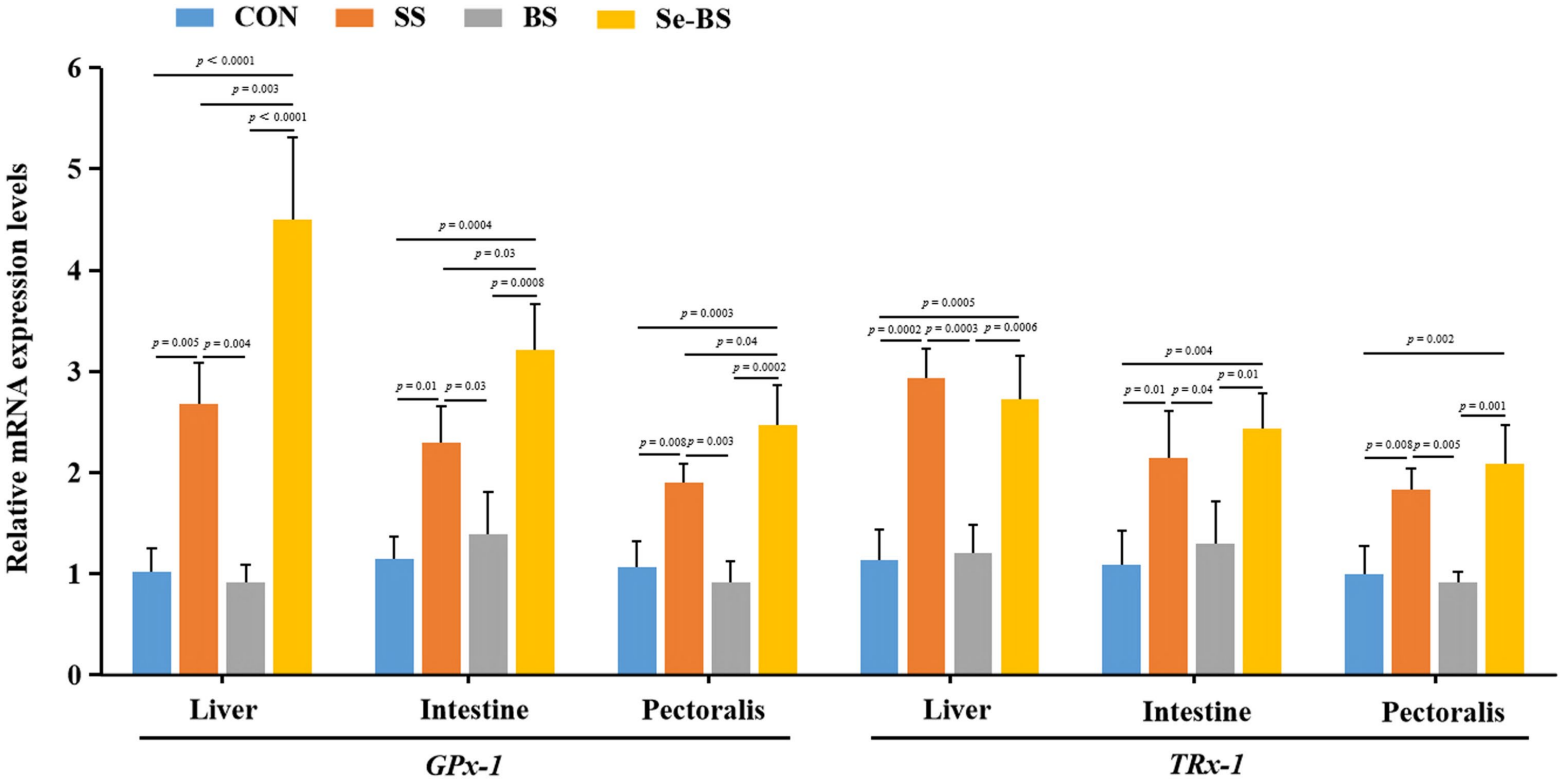
The effects of dietary supplementation with selenium (Se) and *Bacillus subtilis* (BS) on the relative mRNA expression levels of antioxidant genes in liver, intestine, and pectoral muscle. The data are presented as the mean ± standard deviation. *P*-values indicate group differences.

Effects of Se-BS on the relative mRNA levels of antioxidant genes are shown in Figure 5. Compared with the control group, levels of *GPx-1* mRNAs in the liver (*p* = 0.005, *p* < 0.0001), intestine (*p* = 0.01, *p* = 0.0004), and pectoral muscle (*p* = 0.008, *p* = 0.0003) were significantly increased in SS and Se-BS groups. Compared with the BS groups, levels of *GPx-1* mRNAs in the liver (*p* = 0.004, *p* < 0.0001), intestine (*p* = 0.03, *p* = 0.0008), and pectoral muscle (*p* = 0.003, *p* = 0.0002) were significantly increased in SS and Se-BS groups. Compared with the SS groups, levels of *GPx-1* mRNAs in the liver (*p* = 0.003), intestine (*p* = 0.03), and pectoral muscle (*p* = 0.04) were significantly increased in Se-BS group. Compared with the control group, levels of *TR-1* mRNAs in the liver (*p* = 0.0002, *p* = 0.0005), intestine (*p* = 0.01, *p* = 0.004), and pectoral muscle (*p* = 0.008, *p* = 0.002) were significantly increased in SS and Se-BS groups. Compared with the BS groups, levels of *TR-1* mRNAs in the liver (*p* = 0.0003, *p* = 0.0006), intestine (*p* = 0.04, *p* = 0.01), and pectoral muscle (*p* = 0.005, *p* = 0.001) were significantly increased in SS and Se-BS groups.

### 3.4. Effects of Se-BS on the amino acid content

**Figure 6:**
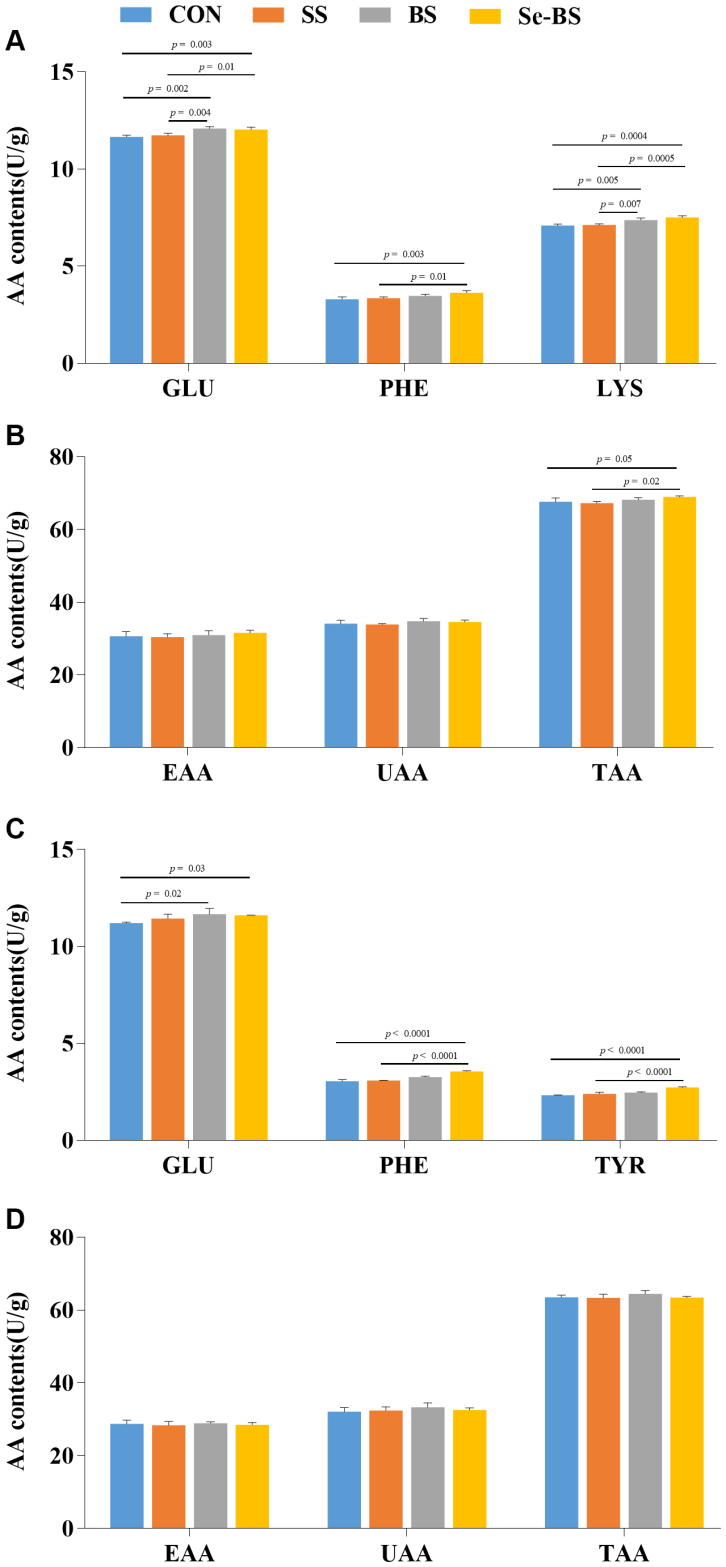
The effects of dietary supplementation with selenium (Se) and *Bacillus subtilis* (BS) on the contents of amino acids in broiler pectoral muscle **(A,B)** and leg muscle **(C,D)**. The data are presented as the mean ± standard deviation. *P*-values indicate group differences.

The effects of Se-BS on amino acid content in the meat are shown in Figure 6. Compared with the control group, contents of GLU (*p* = 0.002, Figure 6A; *p* = 0.02, Figure 6C) in the pectoral and leg muscles were significantly increased by BS treatment. Contents of GLU (*p* = 0.003), PHE (*p* = 0.003), LYS (*p* = 0.0004), and TAA (*p* = 0.05) in the pectoral muscle (Figures 6A,B) and the GLU (*p* = 0.03), PHE (*p* < 0.0001), and TYR (*p* < 0.0001) in the leg muscle (Figure 6C) were significantly increased in Se-BS group. Compared with the SS group, contents of GLU (*p* = 0.004) and LYS (*p* = 0.007) in the pectoral muscle (Figure 6A) were significantly increased in BS group. Contents of GLU (*p* = 0.01), PHE (*p* = 0.01), LYS (*p* = 0.0005), and TAA (*p* = 0.02) in the pectoral muscle (Figures 6A,B), and PHE (*p* < 0.0001) and TYR (*p* < 0.0001) in the leg muscle (Figure 6C) were significantly increased in Se-BS group.

### 3.5. Effects of Se-BS on trace elements

**Figure 7:**
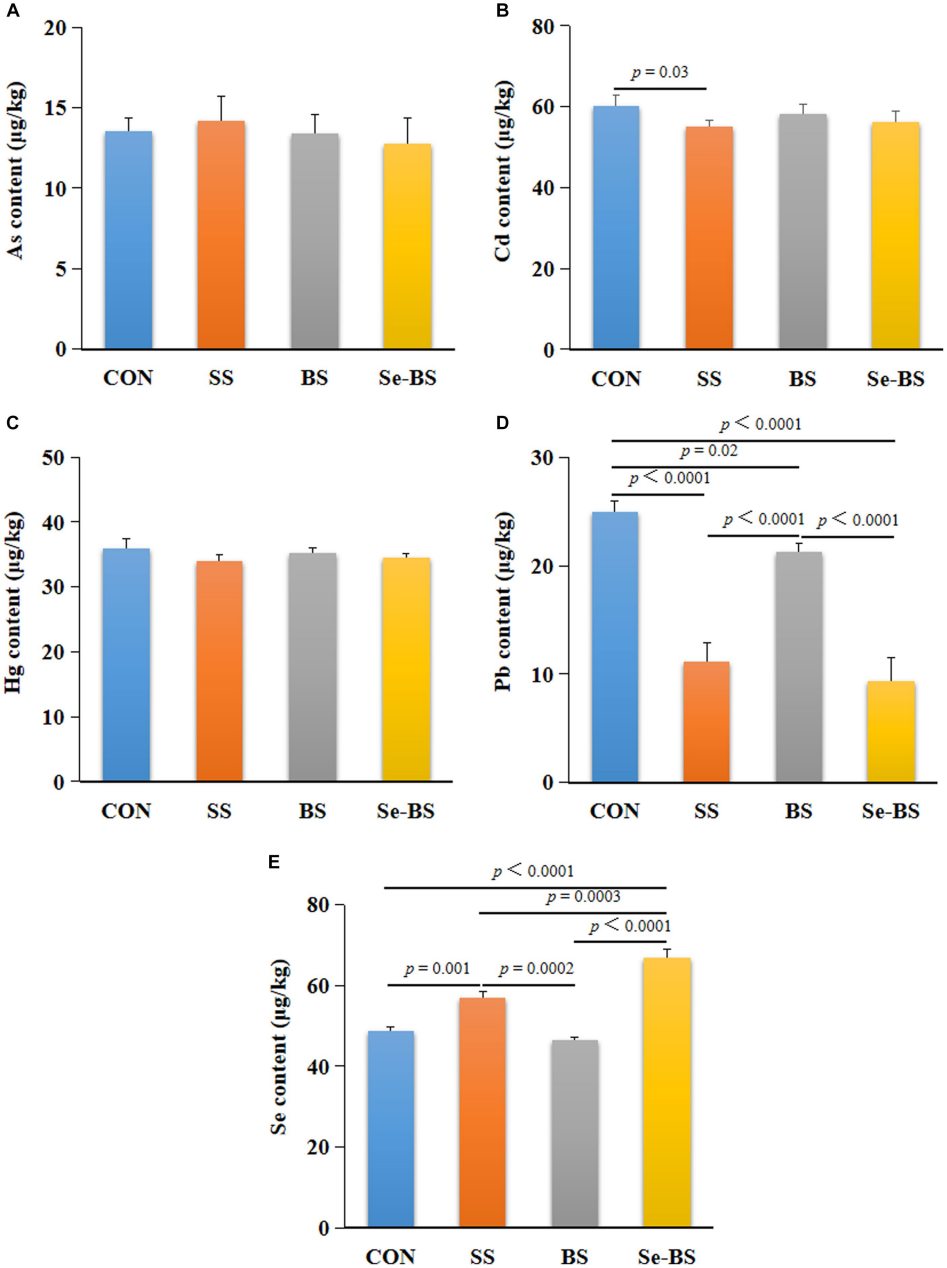
The effects of dietary supplementation with selenium (Se) and *Bacillus subtilis* (BS) on the contents of trace elements in the pectoral muscle of broilers. **(A)** The arsenic (As) content, **(B)** the cadmium (Cd) content, **(C)** the mercury (Hg) content, **(D)** the lead (Pb) content, and **(E)** the Se content. The data are presented as the mean ± standard deviation. *P*-values indicate group differences.

The effects of Se-BS on trace elements in the meat are shown in Figure 7. Compared with the control group, the content of Se (*p* = 0.001, Figure 7E) was significantly increased, and contents of Cd (*p* = 0.03, Figure 7B) and Pb (*p* < 0.0001, Figure 7D) were significantly decreased in SS group. BS significantly decreased the Pb content (*p* = 0.02, Figure 7D), and Se-BS significantly decreased the Pb content (*p* < 0.0001, Figure 7D) and increased the Se content (*p* < 0.0001, Figure 7E) in the meat. Compared with the SS group, BS significantly increased the Pb content (*p* < 0.0001, Figure 7D) and decreased the Se content (*p* = 0.0002, Figure 7E) in the meat, and Se-BS significantly increased the Se content in the meat (*p* = 0.0003, Figure 7E). Finally, compared with the BS group, the content of Se (*p* < 0.0001, Figure 7E) was significantly increased and the content of Pb (*p* < 0.0001, Figure 7D) was significantly decreased in the meat of Se-BS group.

### 3.6. Effects of Se-BS on volatile substances

Effects of Se-BS on volatile substances in the meat are shown in Table 3. Compared with the control group, three types of nitrogenous volatile substances were significantly increased in the meat of SS and BS group, and five types of nitrogenous volatile substances were significantly increased in the meat of Se-BS group. Compared with the SS and BS groups, Se-BS increased two types of nitrogenous volatile substances in the meat.

**Table 3:** The effects of dietary supplementation with selenium (Se) and *Bacillus subtilis* (BS) on the volatile substances in broiler meat.

Items	CON	SS	BS	Se-BS
C_8_H_16_O_2_ (Isoamyl propionate)	+	+	+	+
C_4_H_10_OS (3-Methylthiopropanol)	+	+	+	+
C_10_H_11_NO_5_ (3-(3-carboxyl-4-hydroxy phenyl) -d-alanine)	−	+	+	+
C_4_H_10_N_2_ (2-(aziridin-1-yl)ethanamine)	−	+	+	+
C_2_H_8_O_2_Si (dimethylsilanediol)	+	+	+	+
C_12_H_16_N_4_O_5_ (1-formyl-3-ethyl-6-β-D-trifuranosyl pyrazole [4,5-b] imidazole)	+	+	+	+
C_4_H_8_O_2_ (ALDOL)	+	+	+	+
C_5_H_12_O (3-Methyl-1-butanol)	+	+	+	+
C_16_H_48_O_6_Si_7_ (hexadecamethylheptasiloxane)	+	+	+	+
C_16_H_48_O_8_Si_8_ (hexadecamethylcyclooctasiloxane)	+	+	+	+
C_16_H_24_O_2_ (Ethylhexyl ester p-toluinic acid)	+	+	+	+
C_16_H_22_O_4_ (Phthalic acid diisobutyl ester)	+	+	+	+
C_7_H_15_NO (3,3-Dimethyl-1-(methylamino)-2-butanone)	−	+	+	+
C_6_H_18_O_3_Si_3_ (Hexamethylcyclotrisiloxane)	+	+	+	+
C_4_H_10_OS (3-(Methylthio)-1-propanol)	+	+	+	+
C_4_H_6_N_6_O_3_ (7-nitroamino-3-1-octane-2,4,6, 8-tetrazepidacyclic ring [3,3,0])	−	−	−	+
C_7_H_17_N (5-methyl-2-hexanamine)	−	−	−	+

## 4. Discussion

Carcass quality is affected by genetic and environmental factors as well as nutrition. Researchers have confirmed that the source and level of Se in the diet do not affect the carcass characteristics of broilers. Different organic Se concentrations have no effect on the carcass traits and relative viscera weights of broilers (24, 25). However, Choct et al. (26) found that dietary Se yeast increased visceral weight and pectoral muscle production (26). In the present study, SS and BS had no significant effect on the relative organ weights of broilers, but both treatments improved the carcass weight, the half-eviscerated weight, and the carcass percentage of broilers, respectively. Moreover, Se-BS group had a better slaughter performance than BS and SS, which suggested that Se-BS treatment was more efficient than *Bacillus subtilis* or Se alone in promoting the growth performance of broilers. Therefore, we consider that the combination of Se and BS promotes water and protein deposition in tissues via binding of Se with bacteria.

The physicochemical qualities of meat products, including cooking loss, lightness, color, shear force, dry matter, drip loss, and pH value directly influence the willingness of consumers to purchase meat (5, 27, 28). Researchers have shown that various Se sources and BS strains have positive effects on meat quality (5, 29). More than 80% consumers consider meat color as the primary decision factor for meat consumption. As reported, dietary probiotics could improve chicken meat quality by altering the color (15). The present study demonstrated that supplementation with BS and Se-BS had a better effect on meat color of pectoral muscle. These findings suggest that BS is the main factor for improving meat color. Drip loss is an important examination indicator for moisture loss from chicken meat, and shear force is often known as indicator of tenderness. The two factors are important sensory qualities of meat that influence consumer satisfaction. Studies reported that dietary probiotics exerted beneficial effects on the drip loss and shear force of chicken meat (15). Present study discovered both SS and BS supplementation improved the shear force and drip loss of pectoral muscle, Se-BS showed better shear force and drip loss of meat than SS or BS alone, indicating that the combined application of Se and BS better enhances tenderness and drip loss. Therefore, the physiochemical properties of meat were improved by Se and BS in different ways, but still exert mutually reinforcing effects. Furthermore, combined application of Se and BS produced more desirable effects than either agent alone.

The oxidative status of muscles is closely related to meat quality traits. Therefore, relieving oxidative stress may be a chief way to improve meat quality and to prolong shelf life (30, 31). As reported, the quality of meat can be improved by Se sources and some probiotics in dietary supplementation based on their antioxidant capacity (15, 32). Similarly, we found that organic and inorganic Se as well as BS could significantly improve the antioxidant capacity of the pectoral and leg muscles, including increasing T-AOC, the activities of glutathione system-related enzymes, and decreasing the MDA and GSH level. The enhanced effect of Se on the antioxidant status of muscle can be attributed to the fact that Se is an essential component of GPx, which is the main enzyme of the ubiquitous glutathione antioxidant system in living organisms. GPx can remove excessive reactive oxygen species and reduce the peroxidation of muscle protein and fat. In addition, the levels of *GPx-1* and *TRx-1* mRNAs were upregulated in muscles by Se supplement. This finding suggested that the effect of Se on antioxidant capacity of the pectoral muscle was improved by regulating the expression of antioxidant enzyme genes. Except for improving animal production performance and the antioxidant capacity by inhibiting oxidative stress (32, 33), BS can also protect breast meat against storage-induced oxidative stress by improving the free radical scavenging capacity and antioxidant activity (15). In this experiment, BS increased the activity of antioxidant enzymes and decreased the MDA content. In addition, Se-BS significantly increased the activity of antioxidant enzymes and T-AOC and decreased the MDA content compared with SS and BS alone, indicating that the combination of Se and BS had a synergistic effect on improving meat quality. Moreover, there may be other regulatory mechanisms beyond modulating the glutathione system, but this potentiality requires further investigation.

The types and contents of amino acids, especially EAA and UAA, are evaluated to assess the quality of protein. It is generally believed that the higher the amino acid content in meat, the higher its nutritional value and taste (1, 34). A variety of nutrients can be synthesized by BS, such as proteins, amino acids, polypeptides, and vitamins in the metabolic process of the gastrointestinal tract, and improve the digestibility and absorption of protein in the feed (35, 36). As reported, Se treatment significantly increased LYS and ornithine in plasma of rat (37). Furthermore, supplementing Se significantly alleviated the Cadmium-induced adverse effects on leucine, arginine, valine, and proline in chicken pectoral muscles via analyzing the amino acids profiles (38). Meanwhile, the present study showed that the Se-BS group had higher GLU, PHE, LYS, TYR, and TAA contents in the pectoral or leg muscles, suggesting that the biological activity of BS in the gut might be enhanced by Se, thereby enriching amino acid production in the meat.

Heavy metals and metalloids are well-known and ubiquitous environmental pollutants that threaten food quality and safety, and even the health of animals and humans (39, 40). Researchers have shown that Se has an antagonistic effect on Pb, Hg, and As levels. Dietary Se supplementation can prevent lipid peroxidation, apoptosis, and histopathological changes caused by heavy metals, increase the excretion of heavy metals; and reduce the deposition of heavy metals (41–43). In the present study, both SS and Se-BS supplement increased the Se content and decreased the Pb and Cd contents, but had no effect on As and Hg deposition in the pectoral muscle. Although these results could be related to the broiler strain and the feeding protocol, it is more likely that the heavy metals identified in this study are from the feed given to the broilers. The Pb and Cd contents in the feed are close to the toxic doses, while the As and Hg contents are far from the harmful doses. The results also showed that Se was better at promoting the excretion or reducing the deposition of heavy metals when they are present at toxic doses compared with lower doses. Although the deposition of Pb was reduced by the addition of BS, similarly to a previous study (44). Se-BS further disrupted the deposition of Pb. These results indicated that the combination of Se and BS acts synergistically to promote a stronger antagonistic effect on heavy metal deposition, thus reducing heavy metal-related toxicity. This may be attributed to the ability of Se to antagonize the toxicity of As and Cd by sequestering these elements into biologically inert complexes and/or through the action of Se-dependent antioxidant enzymes (45–47), as well as the biosorption complexation and chelation of BS (40). However, the main mechanism of their synergistic antagonism against heavy metal toxicity requires further investigation.

Volatile flavor compounds are important indicators to evaluate the quality of chicken meat. Although raw chicken meat is not aromatic, some aromatic precursors are produced by fat oxidation, the Maillard reaction, and thiamine degradation after heating, which generates the aroma of cooked meat. Approximately 90% of the aromatic substances are derived from lipid oxidation, followed by the Maillard reaction and thiamine degradation. Although the latter two reactions produce less than 10% of these volatile substances, their influence on meat flavor cannot be underestimated. They mainly produce carboxyl compounds, sulfur compounds, nitrogen, and oxygen heterocyclic compounds. Previous research suggested that the types and contents of volatile compounds could be regulated by organic Se, especially aldehydes and ketones, which affected the changes of muscle flavor (48). In the present study, the C_10_H_11_NO_5_, C_4_H_10_N_2_, and C_7_H_15_NO were detected in the meat by supplementing Se and BS respectively, which indicated that both Se and BS exert biological effects to improve the flavor of chicken meat. In addition, C_4_H_6_N_6_O_3_ and C_7_H_17_N were detected in the meat in the Se-BS group. These changes may be associated with BS metabolism. Se-BS supplementation also promoted an increase in nitrogen-containing heterocyclic flavor compounds in muscles.

## 5. Conclusion

In conclusion, dietary Se-BS supplementation improves slaughter performance, meat quality, and flavor of broiler meat. Se-BS is a better feed additive than BS or SS alone. The findings in this paper provide new insights into the clinical application of Se-BS in diets.

## Data availability statement

The original contributions presented in the study are included in the article/supplementary material, further inquiries can be directed to the corresponding author.

## Ethics statement

The animal study was approved by the Animal Care and Use Committee of Qingdao Agricultural University in accordance with laboratory animal guidelines (GB/T35892-2018, National Standards of the People’s Republic of China). The study was conducted in accordance with the local legislation and institutional requirements.

## Author contributions

JD: Conceptualization, Methodology, Validation, Writing – original draft. HQ: Data curation, Methodology, Writing – review & editing. SG: Writing – review & editing. LH: Data curation, Methodology, Writing – review & editing. HL: Data curation, Methodology, Writing – review & editing. LZ: Methodology, Writing – review & editing. FC: Conceptualization, Data curation, Funding acquisition, Methodology, Writing – review & editing.
